# A Data-Driven Long Time-Series Electrical Line Trip Fault Prediction Method Using an Improved Stacked-Informer Network

**DOI:** 10.3390/s21134466

**Published:** 2021-06-29

**Authors:** Li Guo, Runze Li, Bin Jiang

**Affiliations:** 1 College of Information Engineering, Hubei Minzu University, Enshi 445000, China; gl-hbmzu@hbmzu.edu.cn (L.G.); Lrz-hbmzu@hbmzu.edu.cn (R.L.); 2 College of Automation Engineering, Nanjing University of Aeronautics and Astronautics, Nanjing 211106, China; 3 College of Electronics and Information Engineering, Sichuan University, Chengdu 610065, China

**Keywords:** data-driven, power system, line trip fault, long sequence prediction, stacked-informer networks, gradient centralization

## Abstract

The monitoring of electrical equipment and power grid systems is very essential and important for power transmission and distribution. It has great significances for predicting faults based on monitoring a long sequence in advance, so as to ensure the safe operation of the power system. Many studies such as recurrent neural network (RNN) and long short-term memory (LSTM) network have shown an outstanding ability in increasing the prediction accuracy. However, there still exist some limitations preventing those methods from predicting long time-series sequences in real-world applications. To address these issues, a data-driven method using an improved stacked-Informer network is proposed, and it is used for electrical line trip faults sequence prediction in this paper. This method constructs a stacked-Informer network to extract underlying features of long sequence time-series data well, and combines the gradient centralized (GC) technology with the optimizer to replace the previously used Adam optimizer in the original Informer network. It has a superior generalization ability and faster training efficiency. Data sequences used for the experimental validation are collected from the wind and solar hybrid substation located in Zhangjiakou city, China. The experimental results and concrete analysis prove that the presented method can improve fault sequence prediction accuracy and achieve fast training in real scenarios.

## 1. Introduction

Reliability and stability are the most important aspects to guaranteeing the safe operation of electrical networks and power systems. Early and accurate fault prediction is a valuable and urgent subject in electrical equipment maintenance and power grid operation. An electrical line trip fault usually happens in a power grid system [1,2]; it will produce a long sequence of fault data in the electrical sensor network, eventually cause power outages and economic losses. The aim of fault prediction is to forecast faults that occur in the power system by analyzing historical data, so as to prevent electrical accidents and ensure system recovery.

In the past decades, many studies have been proposed in the power system faults prediction area [3,4,5,6,7,8,9,10,11,12,13,14], such as expert systems [15,16], rough set [17], neural networks [18,19,20,21,22], etc. In general, it is very meaningful to make adequate good use of those electrical measurement data collected by the power station or state grid; this is valuable and first-hand information for improving the fault prediction performance and for ensuring the reliability and stability of power systems. In recent years, many deep neural network (DNN) methods have been applied in power fault prediction [23,24]; the popularly used models are the recurrent neural network (RNN) [23,25] and long short-term memory (LSTM) network [26]. However, these methods have the main drawback that the prediction accuracy will decrease when the long sequence time-series data including a large number of input temporal information is fed into models. In order to address these issues, a data-driven long time-series line trip fault prediction method is used in this paper; it develops an improved stacked-Informer network to capture temporal features of the long sequence input and then achieves a higher prediction accuracy and superior efficiency. The performance of the presented method obviously outperforms the novel developed conventional Informer network and some other models. In fact, the proposed methodology can be applied in real power systems for fault prediction; it helps to improve fault prevention and reduces the losses caused by electrical accidents. The main contributions of this paper are summarized as follows.

(1) The long time-series line trip fault prediction method using the improved stacked-Informer network is adopted; it exploits more comprehensive temporal information of long sequence input measurement data, includes normal and abnormal current and voltage data of power lines, and then predicts the short sequence output fault. This method achieves a superior generalization performance.
(2) The strategy of gradient centralization (GC) technology is introduced and embedded into the optimizer, replacing the original Adam optimizer usually used in the DNN model for a GC+Adam optimization. GC can be viewed as a projected gradient descent method with a constrained loss function; it operates directly on gradients by centralizing the gradient vectors in order to have zero means. This technology improves the training time of the long sequence time-series fault prediction markedly and improves the accuracy and efficiency of the presented methodology.
(3) Real measurement long sequence data is collected by electrical sensors at a wind solar hybrid power station, which is located in Zhangjiakou City, China. The recorded long sequence data is divided into two datasets, and both of them are conducted on the proposed methodology in order to prove a superior performance in real-scenario application.

The rest of this paper is organized as follows. The related work is described in Section 2. Section 3 introduces the architectures of the proposed method for the long sequence time-series line trip fault prediction in a power system. Then, real collected first-hand data is conducted on the presented methodology to validate an effective and efficient performance in Section 3. Finally, the conclusion and prospective work are summarized in Section 4.

## 2. Related Work

**Figure 1:**
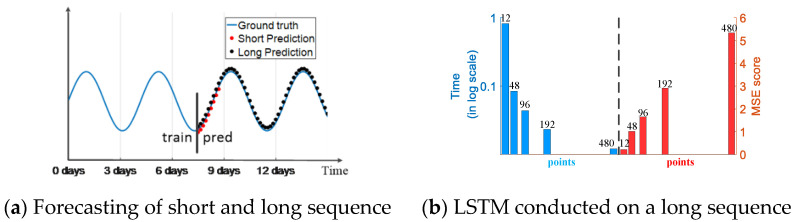
Performance of Informer conducted on a long time-series sequence. (**a**) Sequence forecasting; (**b**) LSTM conducted on a long sequence (here 12, 48 and 96, etc. means points, 12 points is 0.5 day).

Many real-world applications require a long sequence time-series forecasting (LSTF) with a high prediction accuracy, and it has great significances for electrical fault sequence prediction in power systems; this prediction will provide an early alarm to ensure a normal and safe power grid operation. Some models, such as RNN, LSTM and Transformer [27], are good choices for doing a prediction task, but their architecture limits the prediction performance for LSTF. Similar to some illustrations provided by Zhou et al. [28]. Figure 1a indicates that LSTF can extend to a longer period than the short sequence prediction after training process. Figure 1b indicates that when the length of the input long sequence reaches 48, the mean-square error (MSE) value is unacceptable and the inference speed drops rapidly. The architecture of these existing models limits the performance of LSTF, which fails to capture the inherent long-range feature between the output and input.

**Figure 2:**
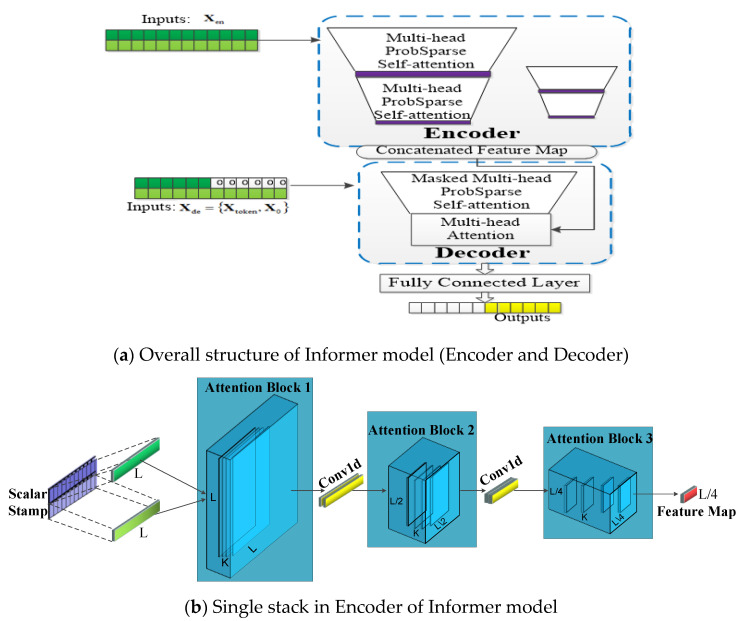
Illustrated architecture of the Informer model. (**a**) is the overall structure of Informer Model; (**b**) is the single stack structure in encoder of Informer model.

In order to deal with this problem, an efficient LSTF transformer-based model named Informer was firstly proposed by Zhou et al. [28] in 2021, which has three special features: (1) a probsparse self-attention block has a good performances on sequential alignment; (2) the self-attention distilling draws the main attention by halving the cascading layer in order to handle long input sequences efficiently; (3) the generative style decoder predicts the long time-series sequence at one forward operation, which drastically improves the inference speed of the long-sequence output. Experiments in Ref. [28] indicate that the Informer model significantly outperforms some existing methods for LSTF. According to Ref. [28], we redraw the main structure of the Informer model and the single stack of the encoder are illustrated in Figure 2a,b, respectively.

The Informer network contains two main parts: encoder and decoder. In Figure 2a, The encoder extracts input massive long sequences Xen (green series, e.g., 1–200 points in the sequence), the self-attention blocks are ProbSparses, which extract the dominating attention and reduce the network size. The decoder receives long sequence inputs X_token_ (e.g., 150–200 points in the input sequence) and the padding element of 0 is X_0_; the concatenated feature map and attention composition are fused to predict the long sequential output instantly (yellow series). Figure 2b represents a single stack of encoder in the Informer network. The horizontal stack stands for an individual one of the encoder replicas in Figure 2a. The input long sequence is L, and K means convolution kernel. Then, the second stack takes half slices of the input L after the convolution, and the subsequent stacks repeat this process. After three layers of attention blocks and two convolution layers, we will obtain a set of L/4 dimension feature maps. The layer stacking replicas increase the robustness. Self-attention distilling and convolution on each layer can get the cascade to decrease, and all stacks’ feature maps are concatenated as the encoder’s output.

## 3. The Proposed Methodology of Long Sequence Time-Series Fault Prediction

Line trip faults are usually existing electrical faults in the power system because of aging, bad insulation and other reasons. It is dangerous to cause massive power blackouts. In general, the recorded electrical measurement data collected by the wind-solar hybrid power station includes the current and voltage during the operating process, and it reflects the occurring faults according to some abnormal fluctuation of data.

### 3.1. Architecture

**Figure 3:**
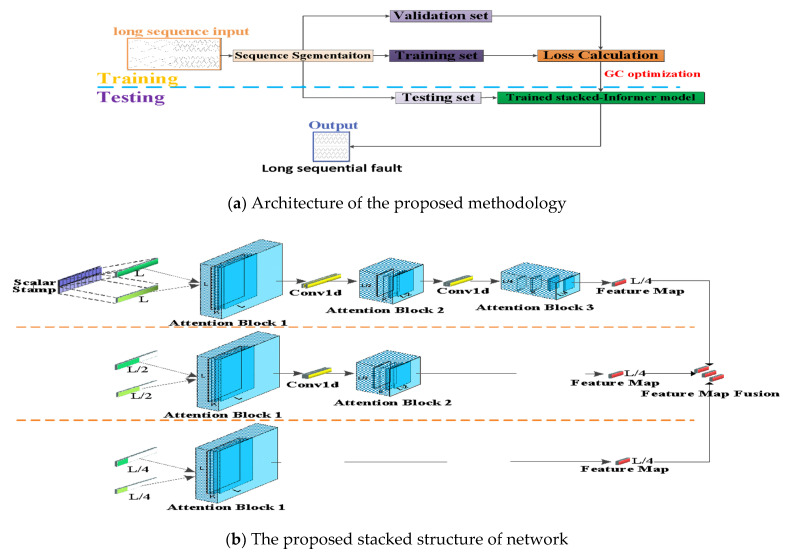
Framework of the proposed methodology and the stacked structure.

In this subsection, the architecture of the proposed methodology is introduced in detail. The presented method is an improved stacked structure based on the Informer network. Besides, the GC technique is introduced in the optimization instead of the previously used Adam optimizer. Figure 3a is the whole framework of the presented methodology, and it contains the training stage and testing stage. The long time-series sequence recorded by the electrical monitoring sensor is the input long sequence. This input sequence is split into three parts of data (short sequences): training set, validation set and testing set. The training set and validation set are applied on the proposed methodology to obtain a trained model with fine-tuned hyperparameters. The trained model is applied on the testing set to obtain the fault sequence prediction. This predicted result is very vital for indicating the operating state so as to remind the worker on duty. Figure 3b is the presented stacked architecture; it is an improved stacked version of the multilayers Informer network. According to the stacked informer structure, suppose the original input long sequence time-series data is L, L is half sliced to L/2 and quartered to L/4, respectively. After that, the input L passes through three attention blocks and two convolution layers, the L/2 long sequence passes through two attention blocks and one convolution layer, and the L/4 long sequence passes through one attention block. Finally, all three L/4 dimensional features are merged into a whole feature map and fed into the decoder. The aim of this stacked structure is to effectively extract the inherent temporal feature of the input long time-series sequence and to improve the robustness of the predicted fault sequence output.

### 3.2. The Improved GC + Adam Optimizer

The optimization technique is very important to effectively and efficiently train a DNN model. Different from most of the adopted optimizers, such as SGD and Adam used in DNN, a new GC technique is embedded into the Adam of the presented methodology. GC technology was firstly proposed by Yong et al. in 2020 [29]; it deals with gradients directly and centralizes the gradient vectors to have zero means, which can be viewed as a projected gradient descent method with a constrained loss function. This technique helps to improve the training process and make it more efficient and stable. We briefly introduce this technique below, and the details are explained in Ref. [29].

Suppose the gradient is obtained by back-propagation; then, for a weight vector *w* whose gradient is $\nabla_{w_{i}}L(i=1,2,\cdots ,N)$, the GC operator denoted by $\phi_{GC}$ is defined as follows:

(1) $$\phi_{GC}(\nabla_{w_{i}}L)=\nabla_{w_{i}}L-\mu \nabla w_{i}L$$

where $\mu \nabla_{w_{i}}L=\frac{1}{M}\sum_{j=1}^{M}W_{i,j}L$, *M* represents the dimension, and *L* is the objective function. The GC formulation in Equation (1) is very simple and efficient, only the mean of the column vectors of the weight matrix is computed, and the mean is removed from each column vector to accomplish the GC technology. We can also rewrite a matrix form of Equation (1) to be Equation (2):

(2) $$\phi_{GC}(\nabla_{W}L)=P\nabla_{W}L,P=I-ee^{T}$$

where *W* is the weight matrix, *P* is the projection matrix in the hyperplane with a normal vector in weight space, and $P\nabla_{W}L$ is the projected gradient [29]. After obtaining the centralized gradient $\phi_{GC}(\nabla_{W}L)$ we can directly use it to update the weight matrix. The technique of GC embedded into Adam is detailed in Ref. [29] (Algorithm 2 in Page 6), so we do not explain it here.

## 4. Evaluated Experiments

In this section, we elaborate the performance of the presented methodology on two real long sequence time-series line trip operating datasets collected by the wind-solar hybrid power station. At first, the data description and experimental settings are detailed in Section 4.1. Then, the performances of the improved GC+Adam optimization and traditional Adam optimizer are discussed in Section 4.2. The overall performance of the presented prediction method is given in the last Section 4.3.

### 4.1. Data Description and Experimental Setting

**Figure 4:**
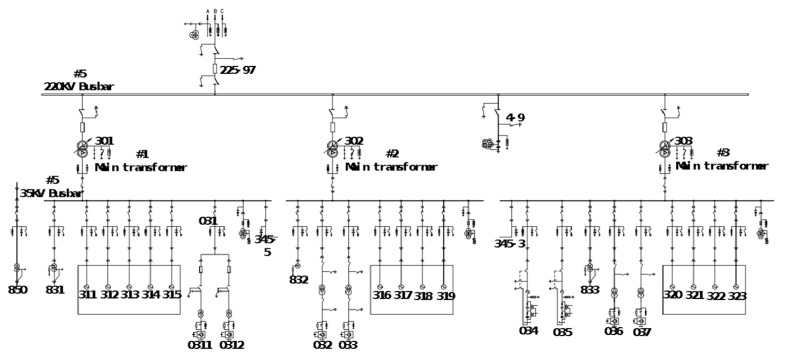
Diagram of the main electrical connection bus line.

**Figure 5:**
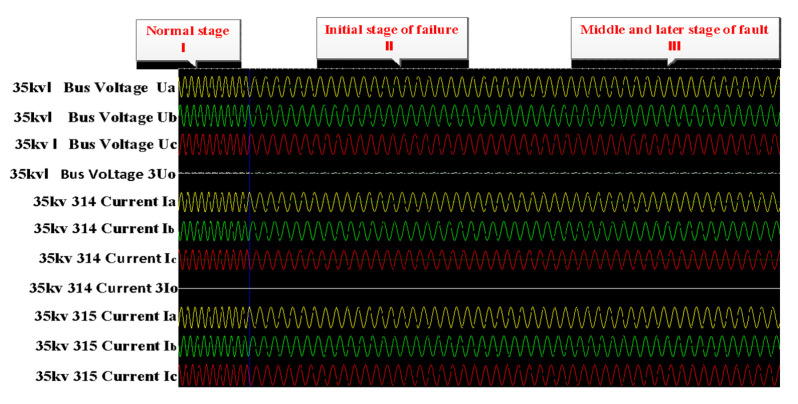
Sampling points (sampling period: five seconds). The left are normal samples, and the right are fault samples.

The real operating data used in the experiments were collected from the wind-solar hybrid substation above 220 KV located in Zhangjiakou city, China. It includes transmission buses, distribution bus lines, and wind-solar hybrid components. The main electrical connection circuit diagram is shown in Figure 4. The electrical measurement data included the three-phase current and voltage values of bus lines, wind motors, circuits, main transformers and capacitors, which were closely related to the faults because of the gradual process of the distribution line. The measurement data was recorded from 10:03:45 a.m. to 10:03:50 a.m. on 17 December 2020, and the sampling period was five seconds. Because the electrical line trip fault happened during a very short moment, the relay protection response unit is us (0.000001 s), which means that the data recording frequency was very high. Although the fault data was only recorded in 5 s, the number could reach up to 13,000 samples. Due to the sudden changes in the current and voltage caused by the line trip fault, the fault-wave recorder records data produced by the electrical sensor in the power system, which is shown in Figure 5. Section 1 represents normal samples recorded in 100 ms by the fault-wave recorder before the fault occurred, and Section 2 is the samples recorded in 2000 ms at the beginning stage of when the fault occurred. The frequency of those two sections is 5000 Hz. Section 3 represents samples recorded in 3 s during the middle and late stages of the failure occurring; the sampling frequency was 1000 Hz. In the early stage of the failure occurring (Section 1), the change is very rapid, so the data sampling frequency is very high (5000 Hz). In the middle (Section 2) and late stages (Section 3) of the recording process, the failure occurs relatively rarely, so the data sampling frequency is low (1000 Hz) so as to reduce the storage cost. We extensively perform experiments on two collected real-world datasets. Dataset 1 is a long time sequence, and it was recorded at 08:17:12:635–08:17:17:736 a.m., on 14 September 2020; the sampling time was 5 s and 101 ms. Dataset 2 is also a long time-series sequence, and it was recorded at 11:36:23:402–11:36:28:503 a.m., on 1 October 2020; the recording time was 5 s and 101 ms. Both datasets contain 13,500 sampling data. All datasets include normal data and fault data in the real sequence, 70% data is used for the training set, 10% of the data is used for the validation set, and the remaining 20% of the data is used for the testing set in our experiments.

For the experimental settings, all of the experiments are conducted on a PC equipped with two GPUs (Nvidia Geforce Titan X × 2ea), Intel Xeon E5-2650 v3 @2.3GHz CPU, and 128GB RAM. The operating system is Windows 10, and the deep learning framework is PyTorch.

### 4.2. Different Optimizer Validation

For the long sequence time-series line trip fault prediction, the major task is to forecast whether there are faults or not during the operation of a power system. The experimental validations of the improved GC+Adam optimizer and original Adam optimizer are discussed in this subsection, respectively. In order to evaluate the performance of the different optimizers, the base architecture selects the original Informer network and the proposed improved stacked-Informer network for an extensive comparison, respectively. All of the parameters are listed in Table 1. For datasets, 13,500 points of the three-phase current and voltage are recorded, and all of those data contain fault data, samples before the line trip fault occurred and normal operation data. The length of the input sequence is 200, and the length of the predicting short sequence is 50. The training loss of the epochs is considered in this experiment, and the training is stopped if the accuracy is stable according to the repeated process.

**Figure 6:**
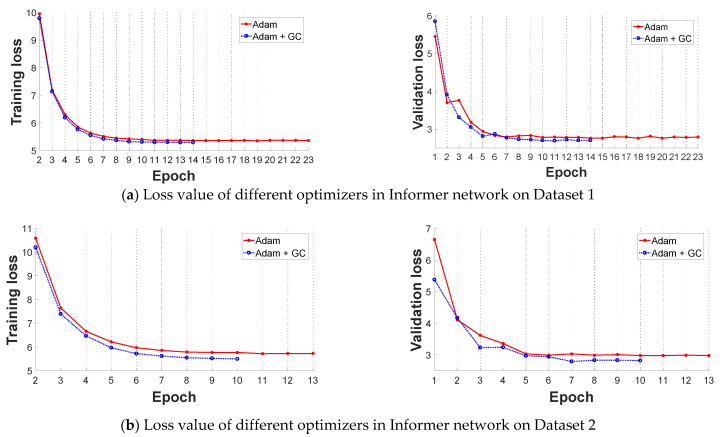
Comparative loss results of the Adam optimizer and improved Adam + GC optimizer in original Informer network (the left is training loss and the right is validation loss). (**a**) Loss value of different optimizers in Informer network on Dataset 1; (**b**)Loss value of different optimizers in Informer network on Dataset 2.

**Figure 7:**
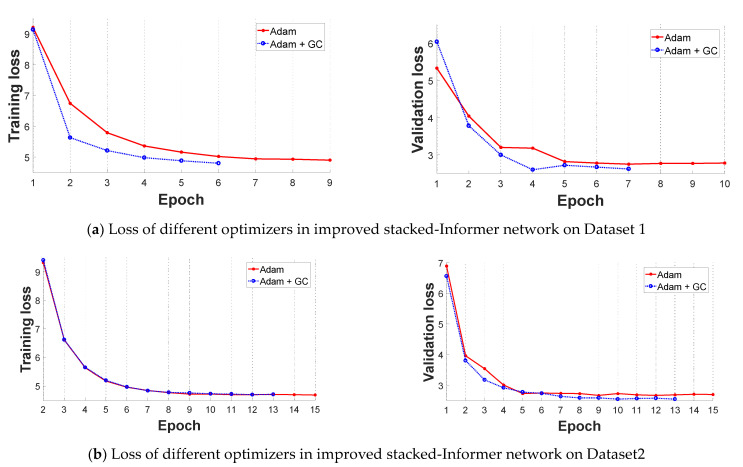
Comparative loss results of improved Adam + GC optimizer and Adam optimizer in the improved stacked-Informer network (the left is the training loss, and the right is the validation loss). (**a**) Loss value of different optimizers in improved Informer network on Dataset 1; (**b**) Loss value of different optimizers in improved Informer network on Dataset 2.

In this subsection, we select the original Informer network and improved stacked-Informer network as the base architecture to evaluate the performance of the improved GC+Adam optimizer and Adam optimizer, respectively. Comparative results are shown in Figure 6 and Figure 7. All of these curves reflect that the training loss is high and unstable at the beginning of the training because the parameters of the networks need to be trained, and the loss value decreases in the latter part. In general, the different base networks with GC+Adam obviously outperform the networks with the original Adam optimizer; it has a faster convergence rate and lower loss value.

### 4.3. Comparative Results of the Presented Method and the Original Informer Network

**Figure 8:**
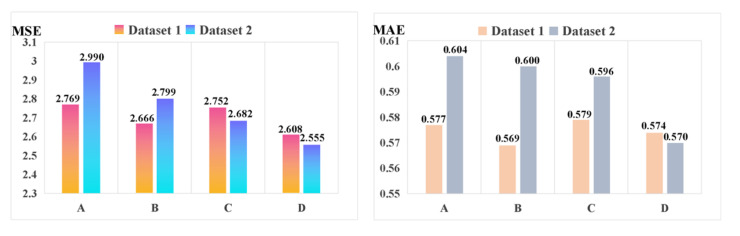
Comparison results of different models for the line trip sequence fault prediction. (Note: A represents Informer with Adam; B represents Informer with Adam + GC; C represents the improved stacked-Informer with Adam; and D represents the improved stacked-Informer with Adam + GC.).

To better explore the superior characteristic of the presented method using the improved stacked-Informer network with the ADM + GC optimizer, it is compared with many other original Informer networks with different optimizers, respectively. For the final fault sequence prediction result, Figure 8 shows the evaluation metric of MSE and MAE on the two provided datasets. We can see that for method D (this represents improved stacked-Informer with Adam + GC), the MSE is 2.55 and the MAE is 0.57; it is obviously lower than the other methods, and this superior performance is improved by the stacked structure and GC optimization technology. The performance of the training time is given in Table 2. We can see that the methods with Adam + GC are obviously more efficient than those with only the traditional Adam optimizer, and the training time of Adam + GC is obviously faster than Adam with the same base network architecture.

### 4.4. Comparative Result of the Different Lengths of the Prediction Output Sequence

**Figure 9:**
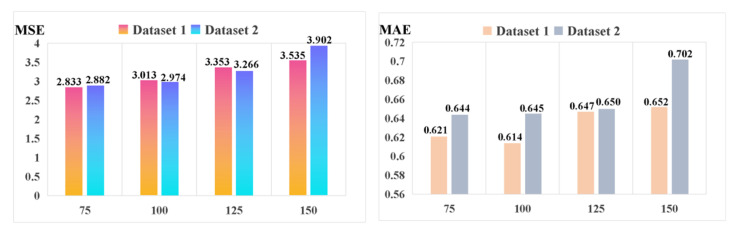
Comparison of the different lengths of the prediction output sequence (improved stacked-Informer with Adam + GC).

In order to further validate the performance of the long time-series fault sequence prediction, we give the evaluated MSE and MAE results in Figure 9, conducted on the proposed method (improved stacked-Informer with Adam + GC) with different lengths of the output prediction sequence. In this experiment, the length of the input long-series sequence is fixed to be 200, and this selection is the same as the experimental setting in the above subsections. From Figure 9, we can see that a longer length of the output prediction sequence will slowly produce an increase in the prediction error, e.g., when the length of the prediction sequence reaches 150, the MSE is 3.535 and 3.902 on Dataset 1 and Dataset 2, respectively. Those values are larger than when the length of the prediction sequence is 75. However, the prediction output does not change suddenly. This phenomenon indicates that a longer fault sequence can be predicted relatively accurately by the proposed method.

## 5. Conclusions

To increase the operational reliability and stability in power systems, a data-driven method for long sequence time-series line trip fault prediction using an improved stacked-Informer network with GC optimization is proposed in this paper. First, two real datasets of three-phase current and voltage values were recorded by electrical sensors during normal operation and a brief occurrence of a line trip fault. The long sequence time-series data is fed into the proposed methodology for the output fault sequence prediction. The stacked structure of the Informer model can improve the generalization ability of the fault prediction, and GC technology increases the training efficiency of the presented method. The experiments were conducted on real data collected from the wind-solar hybrid power substation in Zhangjiakou City, China. Specifically, the corresponding experimental results prove the increasing generalization performance ability. All in all, the improvement of the proposed methodology is noteworthy when compared to the current novel Informer model. In prospective work, we will further explore the performance of different stacked structures of the network and do more comparisons with other outstanding fault prediction methods.

## Figures and Tables

**Table 1:** Parameters in the experiment.

Parameters	Value
Length of input sequence	200
Length of predicting short sequence	50
Batch size	32
Learning rate	0.0001
Dropout rate	0.05
Decay	0.001

**Table 2:** Comparison of different models in training time (s).

Datasets	A	B	C	D
Dataset 1	885.26	538.86	539.23	384.85
Dataset 2	381.52	267.26	572.89	496.34
